# A multi-omics approach to identify molecular alterations in a mouse model of heart failure

**DOI:** 10.7150/thno.68232

**Published:** 2022-01-16

**Authors:** Xiang Zhou, Shuchen Zhang, Yiheng Zhao, Wenjing Wang, Hao Zhang

**Affiliations:** 1 Department of Cardiology, The Second Affiliated Hospital of Soochow University, Suzhou, China.; 2 Thoracic Surgery Laboratory, The First College of Clinical Medicine, Xuzhou Medical University, Xuzhou, China.

**Keywords:** heart failure, multi-omics analysis, scRNA-seq, scATAC-seq

## Abstract

**Rationale:** The morbidity and mortality of heart failure (HF) have been increasing rapidly in recent years. However, the molecular events that link to the phenotype of HF remain unclear. This study aimed to investigate the molecular alterations in the pathogenesis of HF induced by pressure overload.

**Methods:** Transverse aortic constriction was conducted to generate the HF mouse model. A multi-omics study was performed, including integrative analysis of scRNA-seq, scATAC-seq, bulk ATAC-seq and miRNA-seq data. The results of omics analysis were verified by immunofluorescence staining.

**Results:** scRNA-seq analysis identified five major cell types, which exhibits consistency with previous studies. Integrative analysis of ATAC-seq and miRNA-seq showed the alterations of gene expression in HF. Activation of genes involved in immune response at transcriptional level and perturbed expression of their upstream miRNAs confirmed the function of immune cells in the pathogenesis of HF. Analysis of scATAC-seq revealed a NO biosynthetic related gene regulation pattern in endothelial cells of failing hearts.

**Conclusion:** We performed a multi-omics analysis, comparing the transcriptomic, miRNA expression, and chromatin accessibility profile between the HF and control mice, thus providing mechanistic insights into the pathogenesis of pressure overload-induced HF.

## Introduction

With the aging of the population, heart failure (HF) has become a global health concern. Pressure overload is a common cause of HF, which is typically preceded by cardiac hypertrophy, an attempt to counter-balance ventricular wall stress and preserve cardiac function. During the pathological progression from cardiac hypertrophy to HF, various signaling cascades are activated in cardiomyocytes and complex immune responses are triggered in the local tissue 1. Yet, a more detailed atlas of HF needs to be depicted.

Single-cell sequencing is an exquisite and powerful tool for decoding not only the heterogeneity of transcriptome and epigenome, but also the crosstalk and interaction between subsets of cells 2. Wang et al. performed systematic analysis of cellular compositions as well as cell-cell interaction networks and revealed the cellular landscape underlying cardiac function 3. Nomura et al. clarified distinct cardiomyocyte gene programs encoding morphological and functional signatures in cardiac hypertrophy and failure 4. Martini et al. showed the presence of most major immune cell subpopulations in pressure overload-driven HF model 5. More studies are still needed to illustrate how single cardiomyocytes respond to excessive stress and undergo cardiac remodeling toward HF.

Combining single-cell RNA sequencing (scRNA-Seq) with other technologies, such as single-cell ATAC-seq (scATAC-seq) and miRNA-seq, yields more well-rounded and multi-dimensional insights. Chromatin accessibility analysis reveals epigenetic profiling, identifying regions of open chromatin, potential binding sites for DNA binding proteins, as well as nucleosome-bound and nucleosome free positions 6. Here, we performed a multi-omics study on a pressure overload-induced HF mouse model, including integrative analysis of scRNA-seq, scATAC-seq, bulk ATAC-seq and miRNA-seq data, which revealed the novel molecular mechanisms of HF.

## Materials and methods

### Pressure overload-induced HF model

This study was approved by the Animal Ethics Committee of Soochow University. A mouse model of HF was established using the transverse aortic constriction (TAC) method. C57BL/6J male mice, aged 8 to 10 weeks, were randomly divided into TAC group and sham group. After intraperitoneal injection of 1% pentobarbital sodium (40 mg/kg), the mice were fixed on the thermostat in a supine position. A 1.5 cm incision was made at the midline of the sternum and the thymus was separated to fully expose the aortic arch. The 27G needle was ligated with 6-0 silk thread together with the aortic arch, then the needle was quickly extracted and the chest was closed layer by layer. No ligation was performed in the control group, and other procedures were consistent with those in the TAC group. After 4 weeks, the mice were subjected to echocardiography to confirm the success of the HF model. Mice were anaesthetized with pentobarbital sodium and hearts were surgically removed, then left ventricular tissues were separated for subsequent experimental studies.

### Single cell preparation and library construction

Mouse heart tissue was dissociated to single-cell resuspension for the transcriptome sequencing. After rinsed in cold DPBS for three times, the tissue was carefully transferred to digestion buffer (2 mg/ml collagenase I, 1 ml; 2 mg/ml collagenase II, 1 ml; 0.9 U dispase, 0.5 ml; trypsin, 0.5 ml; DMEM, 1.5 ml). The tissue was cut into smaller pieces in the digestion buffer and then gently shook on a 37 ℃ heater shaker for 15 min. The suspension was filtered by a 70 um mesh filter and the cells were concentrated by centrifugation (500g, 5 min at 4℃) and resuspended with 50 to 100 ul DMEM with 10% PBS. The cell concentration was adjusted to 700 to 1200 cells per ul. All the single cell sequencing experiments were performed using the 10X Genomics platform with default protocol. The resuspended cell solution was loaded to the 10X chromium and the GEMs were produced and collected to perform reverse transcription for barcoding, and the first strand cDNA was purified with magnetic beads, all following the Chromium Single Cell 3' Reagent Kits User Guide (v3 Chemistry). After amplification and quantification, the cDNA was used for library construction under standard protocol including size selection, end repair, A-tailing, adaptor ligation, and PCR (10X genomics). The library was sequenced using Illumina NovaSeq platform.

### scRNA-seq data processing, alignment, and clustering

Mapping to mouse mm10 genome, quality control and readcounting of Ensembl genes were performed with cellranger software using default parameters (v2.1.0). Seurat V3 was used to read the expression matrices and for downstream analysis. For quality control of each expression matrix, lowly-detected genes and cells with limited number of genes were discarded from the downstream analysis. The gene filter was set to detection in at least 0.5% of all cells and the cell filter was set to a minimum number of 1000 of total expressed genes per cell. For each expression matrix, the expression value of each gene was first normalized by CPM/100 and then log-transformed (NormalizeData function in Seurat), to generate a normalized matrix ready for integration. Using the variation stabilizing transformation method, the top 2000 variable genes were selected in each matrix and used as input for the 'FindIntegrationAnchors' function of Seurat. The expression matrices were then integrated with the 'IntegrateData' function. The integrated data were dimension reduced with principal component analysis (PCA; top20 dimensions) and then further reduced to two dimensions with uniform Manifold Approximation and Projection (UMAP) which was also used to visualize the clusters. Nearest neighbors were defined among cells with KNN method (FindNeighbors) and cells were then grouped with Louvain algorithm (FindClusters in Seurat, resolution equal to 0.5).

### Using machine-learning methods to annotate cell clusters

Annotation of the clusters was performed by comparing the current dataset with a previously published dataset 7. The cell type annotation of previous study was used and merged into seven major cell types: endothelial cell, fibroblast cell, B cell, Mural cell, T cell, NK cell, glial cell, macrophage, and dendritic cell. A cell type classifier was trained with an R package 'scPred' using 70% of the cells of the reference dataset and the rest 30% were used for testing. A prediction score of 0.8 was used as a threshold for cell type assignment and all the major cell types except NK cells (0.77) could be predicted with an accuracy of higher than 0.9. All the clusters in our current dataset could be solely assigned to one individual cell type except one, which was annotated as cardiomyocytes by markers. The visualization was performed using R package 'pheatmap'. All the annotated clusters were confirmed by detecting the expression of known markers.

### Gene ontology (GO) analysis

R package 'clusterProfiler' was used to compute for the enrichment of GO terms in given gene lists. Among categories of GO terms, we only computed those for 'biological pathways'. Visualization was also performed with the same package using the function 'barplot' implemented in the package.

### Differentially expressed genes (DEGs)

For computing the DEGs of each cluster, only the genes expressed in more than 25% of that cluster were considered, and the expression in all other cells was used as background. For statistical test, we used the 'MAST' method implemented in Seurat. DEGs were defined as genes whose log fold-change were over 0.4 compared to the background, and with a q-value smaller than 0.05.

### miRNA library preparation

Qiagen miRNeasy Mini Kit was used for the total RNA preparation. Briefly, tissue was homogenized in QIAzol Lysis Reagent and chloroform was used to form separated phases. The top layer (water phase) was retained and mixed with ethanol, and then transferred to RNeasy Mini column. The RNA was washed with washing buffer and eluted with RNase free water. Libraries were generated using NEBNext Multiplex Small RNA Library Prep Kit for Illumina sequencing.

### miRNA expression analysis

Raw reads obtained from miRNA sequencing were first processed with adapter trimming using BBDukfrom BBTools (v 37.93). Trimmed reads were mapped to mature miRNAs (Mus musculus) from miRBase database (v 21) via miRExpress (v 2.1.4) to generate miRNA expression profiles. Expression count tables were subjected for downstream analysis in R software (v 3.6.0). The edgeR package (v 3.28.1) was used to identify differentially expressed miRNAs between HF and control groups. Functional analysis was conducted using multiMiR package (v 1.8.0) to predict the targets of miRNAs.

### ATAC-seq library preparation

The ATAC-seq library were prepared according to the OMNI-ATAC protocol 8. Homogenization was applied to break the tissue and obtain purified nuclei through centrifugation. The collected nuclei were subjected to Tn5 enzyme transposable reaction (Omni-ATAC reaction mix) to have fragmentation upon open-chromatin regions. The product DNA was purified (QIAGEN MinElute) and PCR was performed to amplify the fragmented DNA. Cleaned up libraries were size-selected and then subjected for next-generation sequencing (Illumina NovaSeq platform).

### Data analysis of ATAC-seq

Adapters were identified and trimmed with TrimGalore (v 0.6.5), followed by sequence alignment with STAR (v 2.5.4) to the mouse genome (mm10). Sorted bamfiles were subjected to peak calling using MACS2 (v 2.1.2), and the output narrowPeak files from individual samples were used for downstream analysis. To identify consensus peaks, we generated Granges and isolated the peaks that were present in at least three HF and control samples respectively via R packages namely GenomicRanges (v 1.38.0) and rtracklayer (v 1.46.0). The consensus peak lists were annotated with ChIPseeker (v 1.22.1) to identify genes with open promoter regions, and to perform data visualization. The promoter regions were defined as upstream 500bp to downstream 500 bp from the transcriptional start sites. For visualization of ATAC-seq results, average coverage from HF and control samples were determined using normalized bedGraph files and plotted over the genomic region of specific genes using Gviz (v 1.32.0). GO analysis was performed to identify the enriched biological processes for DEGs.

### Cryo-sectioning and immunofluorescent staining

The myocardial tissues were sliced into 5 μm-thickness cryosections and stored at -20℃. For immunofluorescence, the slices were fixed by 4% paraformaldehyde for 15 min, permeablized with 0.5% Triton X-100 for 20 min and blocked by 3% bovine serum albumin for 1 h. The sections were incubated with the primary antibodies overnight at 4℃ and then incubated with the second antibodies at room temperature for 2 h. All the antibodies were purchased from Abcam (Cambridge, MA, USA). The slices were then observed under Zeiss confocal microscope (LSM880). To quantify the immunofluorescent imaging, we used ImageJ to calculate the mean grey value (IntDen/Area) of the measured region.

### scATAC-seq analysis

Raw scATAC-seq data were mapped to the mm10 genome reference by the 'cellranger-atac' tool. The fragment files generated by 'cellranger-atac' were used as input for the R package 'archR'. Cells with less than 4000 unique fragments or transcriptional start site (TSS) enrichment score less than 4.5 were removed. Valid cells passing the filters were clustered by the 'Seurat' method provided by the 'archR' package. UMAP (with 30 neighbors) was chosen for dimension reduction and visualization. The 'addGeneIntegrationMatrix' function from 'archR' was used to map the known cell clusters identified by scRNA-seq. The cluster annotation was further validated by cell type-specific markers. The following analysis of motif enrichment and pair-wised differential analysis were also performed with the recommended settings of 'archR' package.

### Fluorescent *in situ* hybridization (FISH)

The FISH assay was conducted to detect the expression of mmu-miR-3473a and Rora in cardiac fibroblasts (CFBs). After pre-hybridization, cells were incubated with probes (GenePharma, Suzhou, China) in hybridization buffer. DNA was stained with DAPI and the signal was measured using an inverted fluorescence microscope (Nikon, Tokyo, Japan). The sequences of probes are as follows: mmu-miR-3473a, 5'-TGCTGAGCCAT CTCTCCA-3'; Rora, 5'-TCTGAGAGTCAAAGGCACGGCACATCCTAATAAAC-3'.

### Statistical analysis

Statistical analyses were performed using SPSS version 20.0 software. Data were expressed as mean ± SD. The differences between the two groups were compared using the Student's t-test. A p value < 0.05 was considered statistically significant.

## Results

### scRNA-seq was performed on cardiac tissue of HF and control mice

Single-cell transcriptomics profiling was conducted on myocardial tissues of HF and control mice (n = 5 for each group). After quality control, 23033 cells were retained, with 12108 from the control group and 10925 from the HF group. Cell numbers from each of the ten individual samples range from 1926 to 3124. An average of 1510 expressed genes and 3295 unique molecular identifiers were detected in each individual cell.

**Figure 1:**
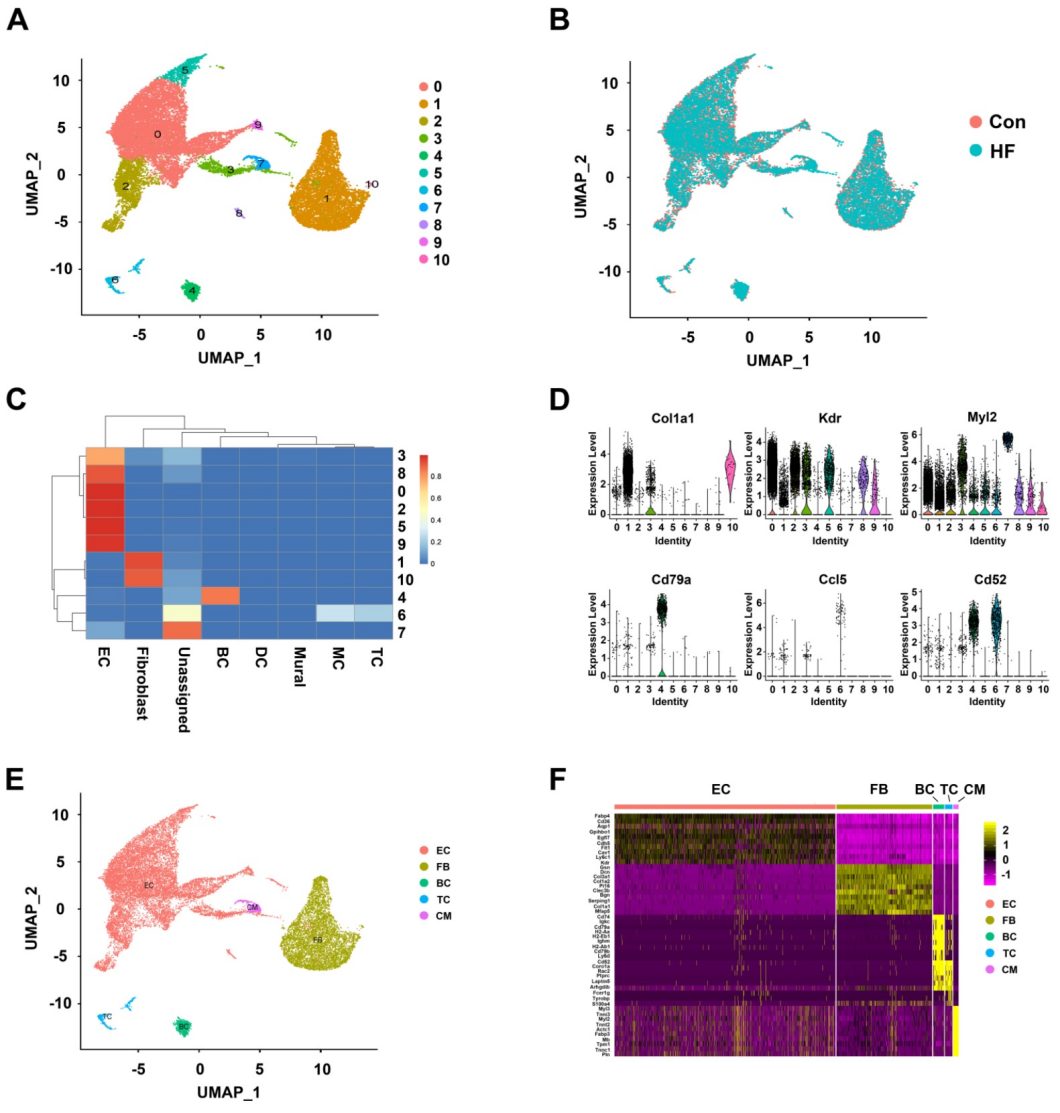
** Clustering and annotation of single cells in control and heart failure (HF) mice. (A)** Initial clustering of 23033 single cells from mouse heart tissue in both control and HF groups visualized in UMAP dimension reduction. **(B)** Overlapping of cells from HF and control groups visualized in UMAP dimension reduction. **(C)** Heatmap of prediction scores matching each cluster of the current dataset to published mouse single-cell profile. **(D)** Verifying the cell type annotation by visualization of known markers. **(E)** A UMAP dimension-reduced view of annotated major cell types. **(F)** The top differentially expressed genes for each cell type visualized in heatmap. EC: endothelial cell; FB: fibroblast; BC: B cell; TC: T cell; CM: cardiomyocyte.

The merged dataset was dimension-reduced using unbiased PCA (with top 2000 highly variable genes as input) and the top 20 principal components were further reduced to two using UMAP (**Figure 1A**). Eleven initial clusters were identified in our dataset. As shown in **Figure 1B**, all the clusters were contributed by both HF and control groups and with good overlapping, indicating minimal batch effect in clustering. In order to annotate the cell clusters and compare the current dataset with previous ones, we applied a machine learning approach named 'scPred' to train a classifier using the published dataset as reference. Nine of the eleven clusters can almost be solely matched to a cell type in the reference dataset (**Figure 1C**). Cluster 7 was labeled as unassigned because it showed low similarity with all the reference data while cluster 6 showed similarity to macrophage and T cell. By checking the expression of known markers (Col1a1 for fibroblast, Kdr for endothelial cells, Cd79a for B cells), we first validated the assignment of cluster 0 to 5 and 8 to 10. We then annotated cluster 6 as T cells with the expression of Ccl5 and Cd52. Moreover, we found that cluster 7 was cardiomyocytes with the expression of Myl2 (**Figure 1D**). The low-dimension embedded cells with complete annotation can be visualized in **Figure 1E**. Differential expression analysis was performed for each cell type and the top DEGs were visualized in heatmap (**Figure 1F**). Full DEG lists were used for GO enrichment and expected biological process terms were enriched in cell types (**Figure S1**).

### scRNA-seq revealed changes in the proportion of immune cells in failing hearts

**Figure 2:**
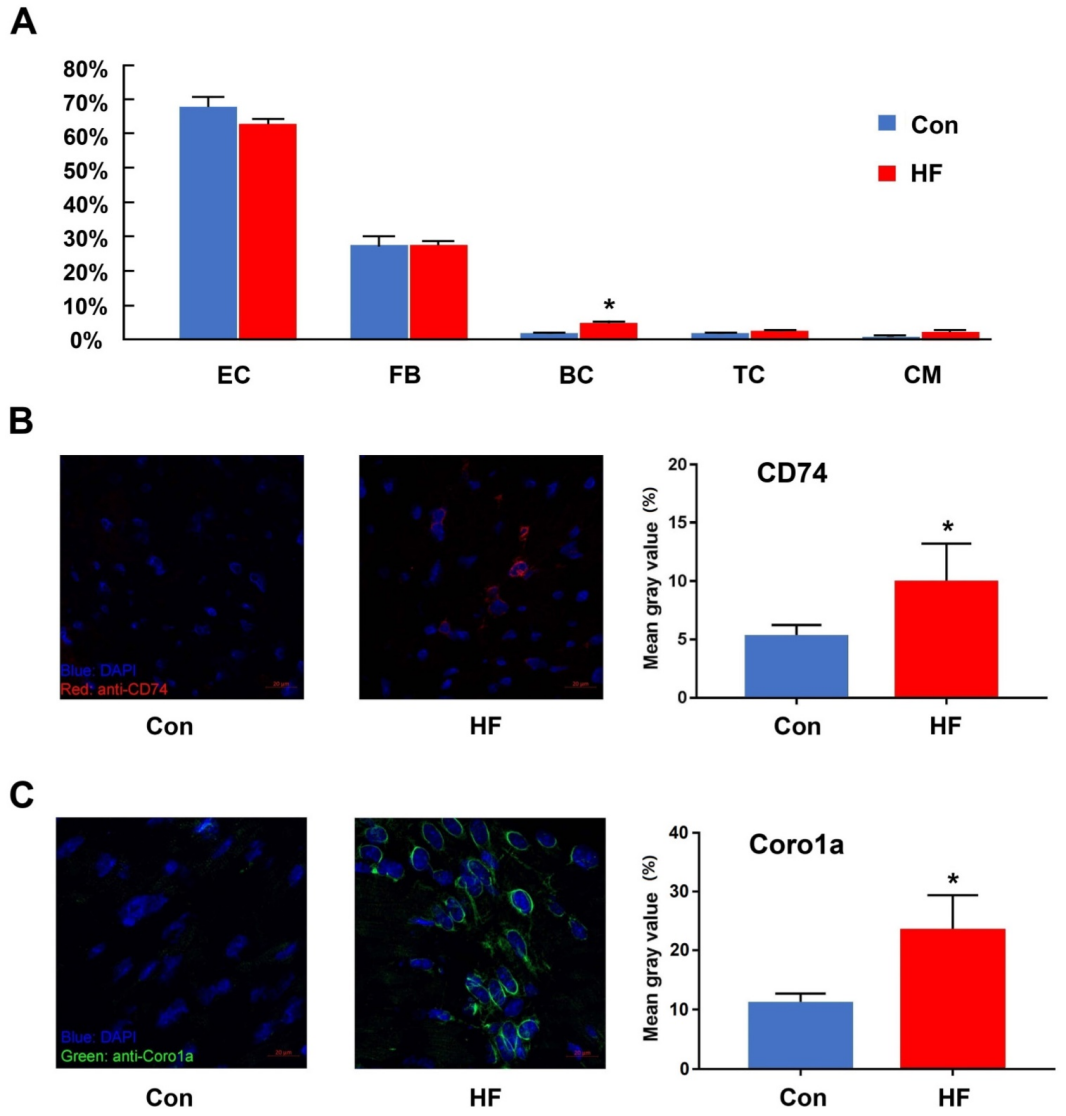
** The changes in the immune cells between control and heart failure (HF) mice. (A)** The proportion of each cell type in control and HF groups. **(B, C)** Immunofluorescent staining and quantitative analysis for CD74 and Coro1a in control and HF groups. (n = 5, *p < 0.05). EC: endothelial cell; FB: fibroblast; BC: B cell; TC: T cell; CM: cardiomyocyte.

One important benefit gained from scRNA-seq is that cell proportion could be quantified and compared among various conditions. Here, we compared the proportion of cell types in myocardial tissue between the HF and control groups (**Figure 2A**). Only B cells showed a 2.7-fold increase in the cell proportion of the HF group. T cells also showed an increased tendency but did not have statistical significance. Since both cell types only had limited overall proportion in the whole population, we assumed that even random sampling during the single-cell preparation may affect their proportion in the final dataset. Therefore, we performed validation experiments with immunofluorescent staining. CD74 was used to stain T cells and Coro1a was used to stain T/B cells. Both staining showed that the immune cells had much higher proportion in the failing hearts (**Figure 2B-C**).

### miRNA-seq analysis identified potential upstream regulators of DEGs

**Figure 3:**
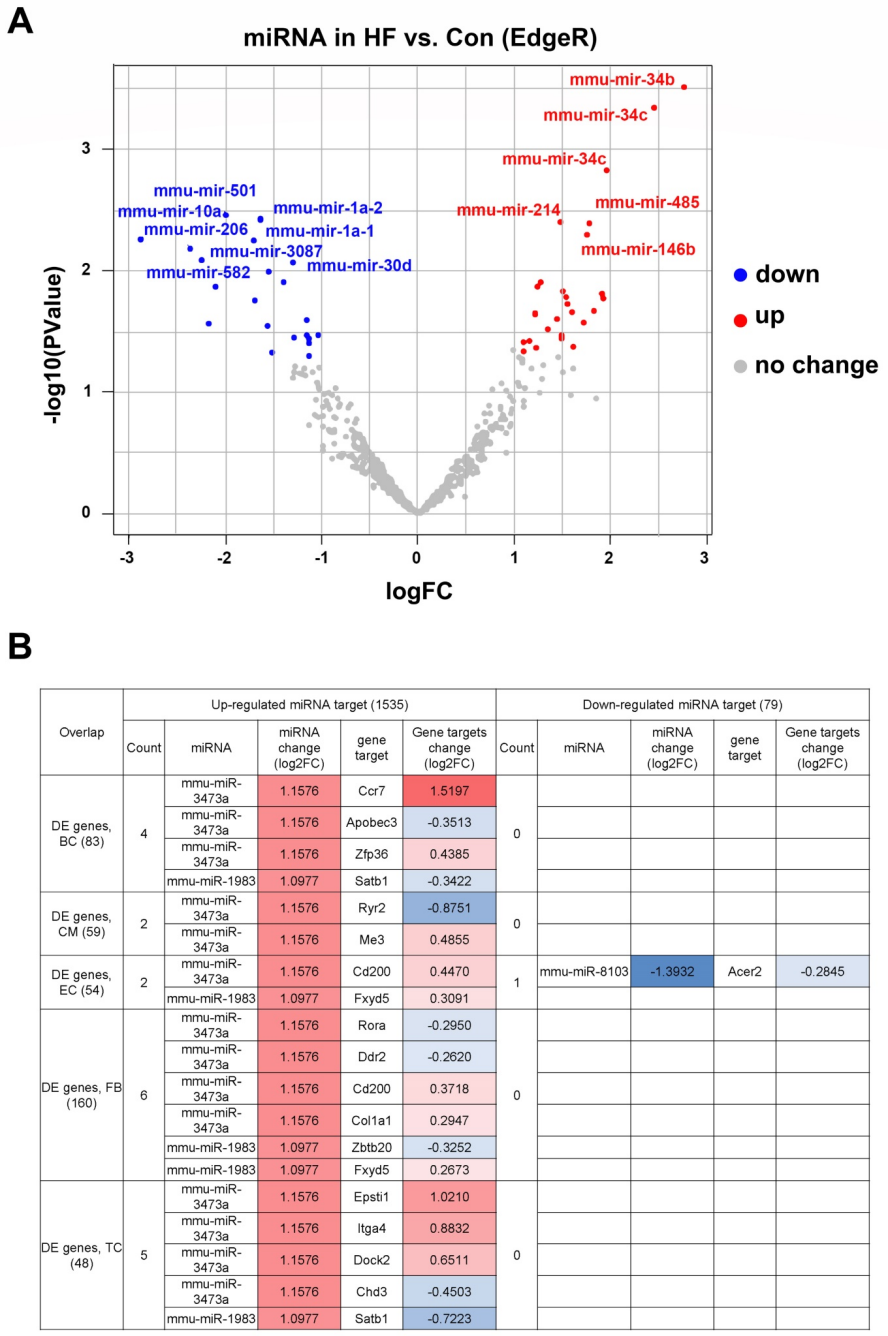
** Differentially expressed miRNAs and corresponding downstream targets. (A)** Volcano plot of differentially expressed miRNAs in heart failure (HF) and control mice (p < 0.05 and absolute log2FC ≥1). **(B)** The downstream targets of above miRNAs overlapped with the differentially expressed genes identified from scRNA-seq analysis.

To determine the upstream regulators of DEGs in myocardial tissue, we performed miRNA-seq in the HF and control groups (**Figure S2**). Our results indicated that 38 miRNAs were up-regulated for at least two-fold in the HF group, whereas 22 miRNAs were significantly down-regulated (**Figure 3A**). The downstream targets of the above miRNAs were highly enriched for genes involved in the development of various organisms, suggesting perturbed development process in HF mice. By combining miRNA-seq with scRNA-seq analysis, we found that a subset of DEGs in various cell types were among the predicted targets of the up-regulated miRNAs (**Figure 3B**). Interestingly, most of the identified targets were under the control of mmu-miR-3473a and mmu-miR-1983, suggesting that these two miRNAs may be the master regulators of HF development.

**Figure 4:**
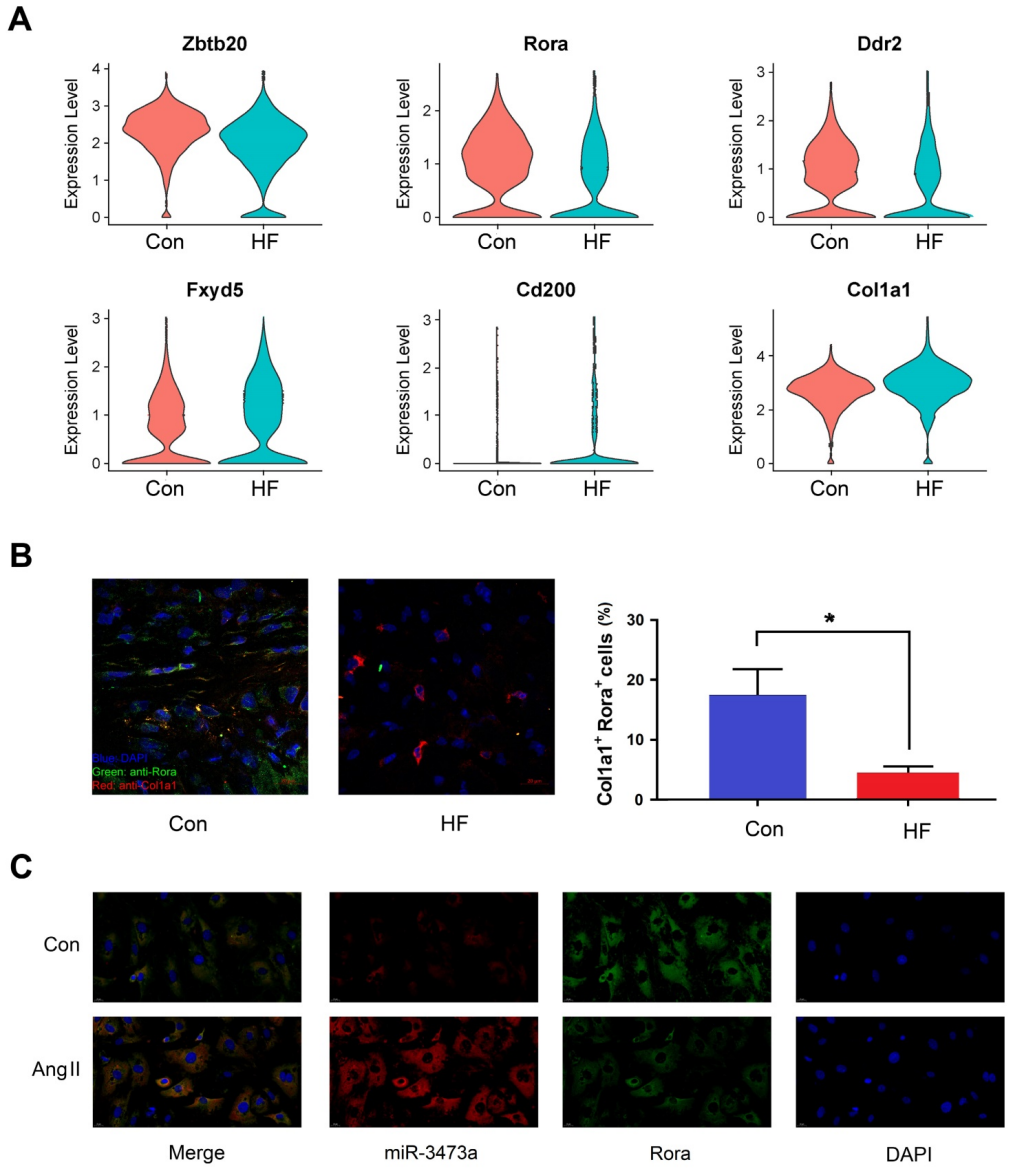
** The miR-3473a/Rora regulatory pathway in cardiac fibroblasts. (A)** Differentially expressed genes in fibroblast cells overlapping with the potential targets from miRNA profiling between control and heart failure (HF) groups, visualized in violin plot. **(B)** Immunofluorescent staining and quantitative analysis of Rora in control and HF mice (n = 5, *p < 0.05). Col1a1 was used to label fibroblast cells. **(C)** FISH experiments were performed to confirm the interaction between miR-3473a and Rora in mouse cardiac fibroblasts treated with Ang II (1 μg/ml).

Differential expression analysis was also performed between the HF and control mice within each cell type. Six genes were found to be differentially expressed in fibroblast cells and identified as targets of miRNAs mentioned above (**Figure 4A**). Among them, we selected *Rora* for further research. Our findings showed that *Rora* was down-regulated in fibroblast cells of HF mice, while its potential regulatory miRNA was up-regulated, indicating a negative regulation from miRNA to gene expression. We then performed immunofluorescent staining on cryosections of myocardial tissue and found that *Rora* was down-regulated in CFBs of HF mice (**Figure 4B**). In order to demonstrate the interaction between mmu-miR-3473a and *Rora*, we conducted FISH experiments in mouse CFBs (**Figure 4C**). As expected, mmu-miR-3473a and *Rora* colocalized in CFBs, indicating mmu-miR-3473a might act as a regulator of *Rora*. Moreover, we noticed a profound down-regulation of *Rora* in CFBs treated with Ang II, while mmu-miR-3473a level was significantly increased.

### ATAC-seq revealed gene expression changes in the development of HF

**Figure 5:**
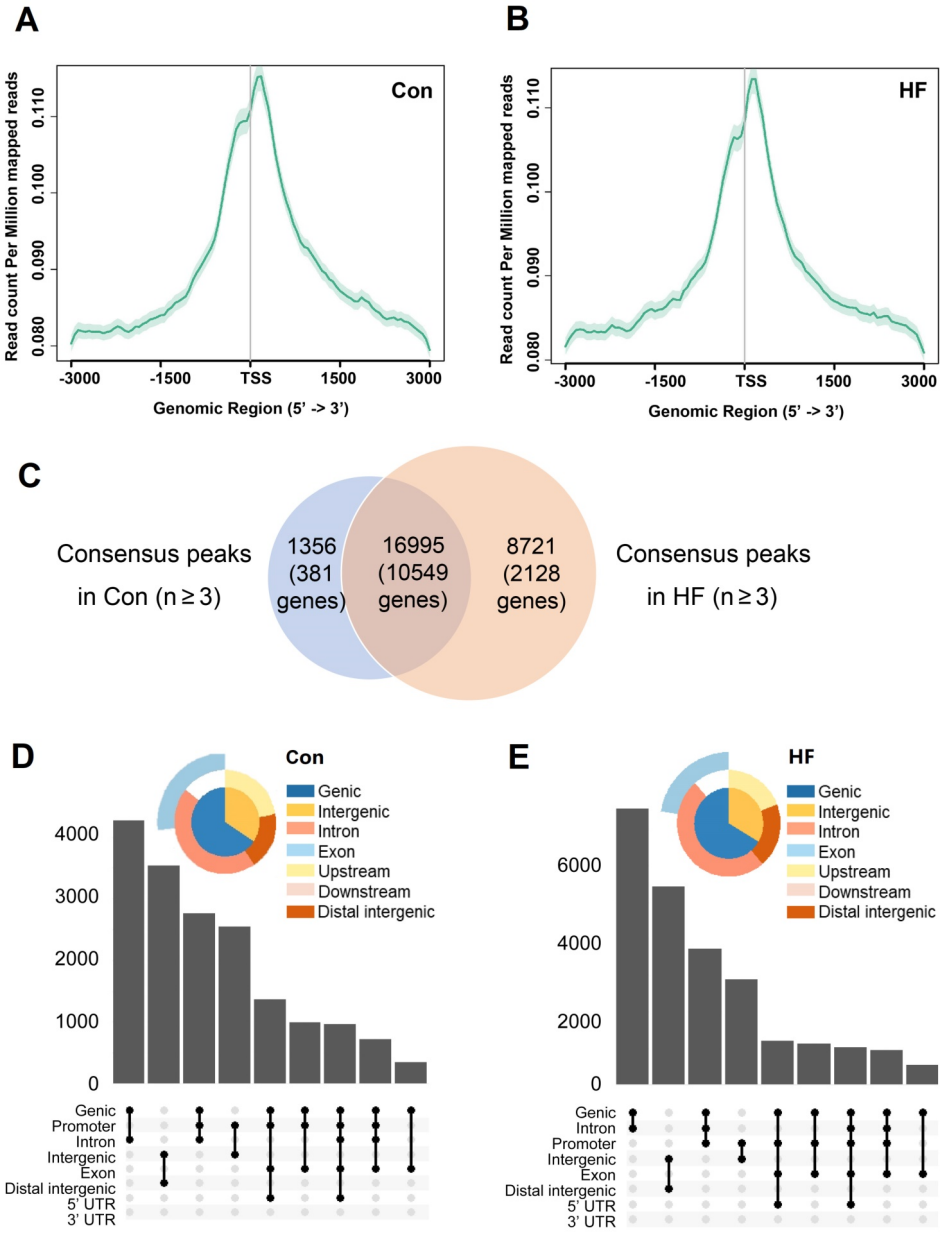
** ATAC-seq analysis and the accessible chromatin regions. (A, B)** TSS enrichment of the ATAC-seq for open-chromatin regions. Genes with accessible chromatin regions from control and heart failure (HF) mice were first determined by the consensus identification. The TSS regions were then merged and plotted among samples of each group. **(C)** Common and different accessible chromatin regions were identified by ATAC-seq in control and HF mice. **(D, E)** Annotated regions and distribution of the accessible chromatin regions in control and HF mice were clustered around the promoter regions.

To confirm whether chromatin remodeling occurs in pressure overload-induced HF and to identify downstream gene targets, we performed ATAC-seq in control and HF mice. Sequenced reads were enriched around TSS for genes with accessible chromatin regions and have similar intensity of coverage between control and HF groups (**Figure 5A-B**). A total of peaks corresponding to 10930 genes were identified in at least 3 control samples, while a substantial increase of accessible chromatin regions was observed in at least 3 HF samples with 12677 genes included (**Figure 5C**). Notably, most of open chromatin regions in the control group remained to be accessible after TAC treatment. Moreover, annotated regions and distribution of the accessible chromatin regions in control and HF mice were clustered around the promoter regions (**Figure 5D-E**). Thus, our findings indicated that pressure overload could induce a global epigenetic change, which is likely associated with the gene expression changes involved in the development of HF.

**Figure 6:**
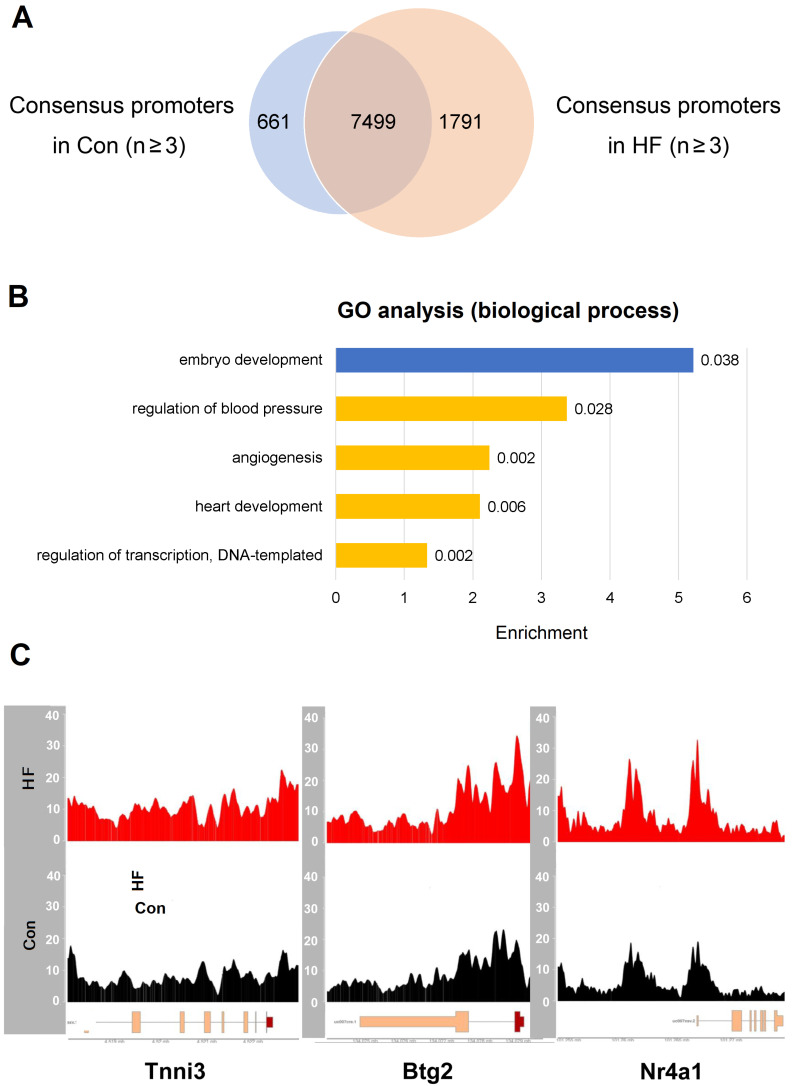
** ATAC-seq revealed gene expression changes in failing hearts. (A)** Common and different accessible promoter regions were identified by ATAC-seq in control and heart failure (HF) mice. **(B)** Gene ontology analysis revealed enriched pathways for the genes with open promoters in HF group but not in control group (yellow), and vice versa (blue). **(C)** HF samples have enhanced ATAC-seq signals at the promoter regions of Tnni3, Btg2 and Nr4a1.

Given that meaningful changes of open chromatin usually reside in the promoter regions which thereby result in perturbed gene expression, we focused our analysis on the genes with accessible promoters in HF samples but not in the controls (**Figure 6A**). For 1791 genes with open promoter regions only in HF samples, the top enriched biological processes include regulation of blood pressure, angiogenesis, heart development and transcriptional regulation (**Figure 6B**). Particularly, we observed enhanced signals from ATAC-seq in HF samples at the promoters of marker genes involved in heart development and cell cycle regulation, including *Tnni3*, *Btg2,* and* Nr4a1* (**Figure 6C**). Multiple other genes showed increased expression in individual cell types based on scRNA-seq data, suggesting consistency with the ATAC-seq results (**Figure S3**). Therefore, the chromatin accessibility effects in pressure overload-induced HF are truly derived from the disease status and effective in driving altered gene expression.

### scATAC-seq revealed the underlying genomic regulation in failing hearts

**Figure 7:**
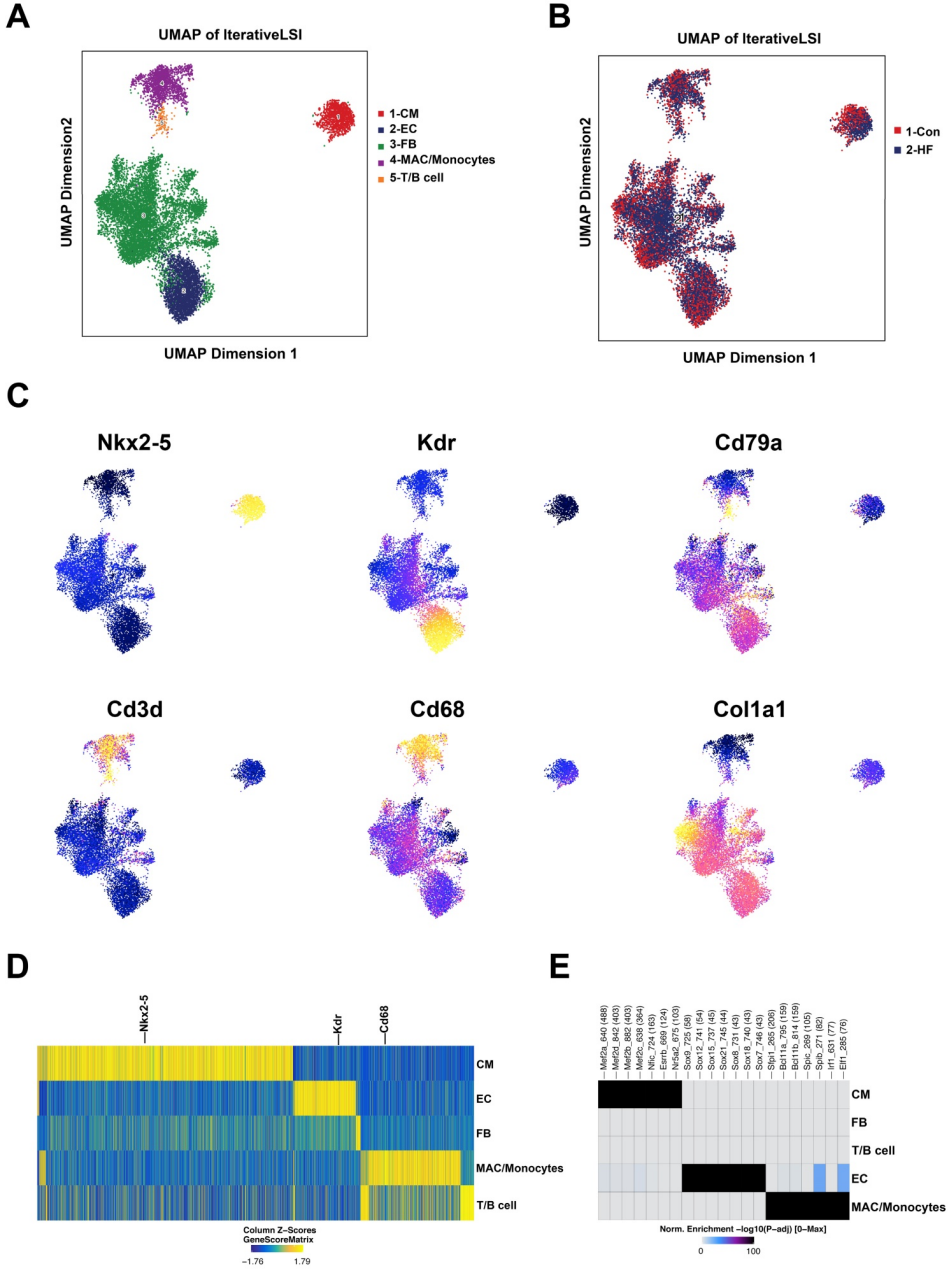
** scATAC-seq profiling of heart tissue in control and heart failure (HF) mice. (A)** Five major cell clusters were identified by scATAC-seq in myocardial tissue. **(B)** UMAP embedding showing the cells from control and HF groups. **(C)** UMAP embedding showing the gene scores of cell type-specific marker genes. **(D)** Heatmap plot of cluster specific marker genes. **(E)** Enriched motifs of the chromatin open regions for the major cell clusters.

scATAC-seq was conducted to investigate the potential genomic regulation during pressure overload-induced HF. With default quality control filters, we obtained 4509 and 8715 valid cells from the control and HF groups, respectively (**Figure S4**). By integrating published scRNA-seq data, we were able to annotate the cell clusters into five major groups: cardiomyocytes, endothelial cells, fibroblasts, macrophages/monocytes and lymphoid cells (**Figure 7A-B**). The annotation of major cell clusters was validated by the gene score of cell-specific markers such as Nkx2-5, Kdr and Cd68 (**Figure 7C-D**). Additionally, the enriched motifs of chromatin open regions for the major cell clusters are shown in **Figure 7E**.

**Figure 8:**
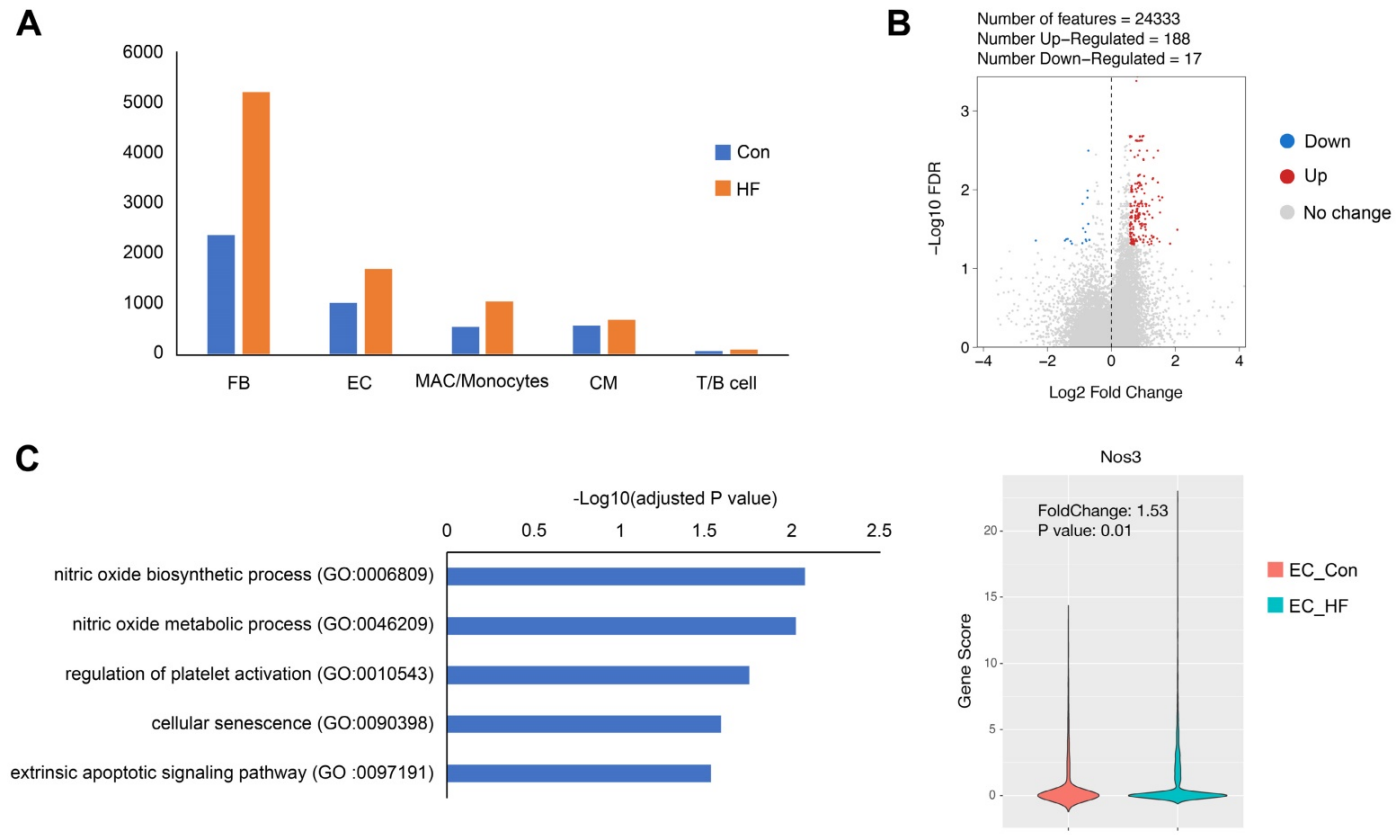
** Gene expression changes in endothelial cells (ECs) of failing hearts. (A)** Bar plot showing the cell numbers of different cell populations in control and heart failure (HF) mice. **(B)** Volcano plot showing the differentially expressed genes in ECs. **(C)** Gene ontology analysis of the up-regulated genes in ECs and the gene scores of Nos3 in control and HF groups.

When comparing the failing hearts with normal controls, we observed the influx of fibroblasts and immune cells, which are consistent to previous studies (**Figure 8A**). More interestingly, we were able to identify 3 times more scATAC peaks and higher fraction of promoter peaks in endothelial cells of HF mice (**Figure S5**). Furthermore, we identified 188 up-regulated and 17 down-regulated genes in the endothelial cell cluster (**Figure 8B**). The up-regulated genes were significantly enriched to nitric oxide (NO) biosynthetic process, regulation of platelet activation, and cellular senescence. It is noted that Nos3, also known as endothelial NO synthase, was significantly up-regulated in pressure overload-induced HF (**Figure 8C**).

## Discussion

In this study, we performed a high-resolution and in-depth transcriptome analysis of myocardial tissue in failing hearts using single-cell sequencing technology from 10X Genomics. Chromatin accessibility alteration and potential miRNA regulation in HF mice were detected by scATAC-seq and miRNA-seq analysis. Combining and cross-validating the above data, we further depicted the atlas of HF and the concomitant immune response in a multi-dimensional way. Among the sequenced 23033 cells, not only major cardiac cell types were identified, but also subpopulations of fibroblasts, endothelial cells and various immune cells 9. More importantly, scATAC-seq further revealed a significant transcriptional reprogramming in the development of HF. NO biosynthetic pathways were found to be activated in the endothelial cells of HF mice. These results provided mechanistic insights into the pathogenesis of pressure overload-induced HF.

Analysis of miRNA-seq data identified mmu-miR-3473a and mmu-miR-1983 as master regulators of heart development. Integrating the miRNA-seq and scRNA-seq data, several genes were found as both up-regulated miRNA targets and DEGs. Retinoid-related orphan receptor-α (RORα) is a member of the nuclear receptor superfamily and plays important roles in alleviating oxidative stress and inhibiting inflammatory response. Using a rupture-prone vulnerable plaque model, Ding et al. verified that melatonin-RORα axis plays a vital role in regulating macrophage polarization via the AMPKα-STATs pathway. RORα could mediate the suppressive effect of melatonin on the differentiation of intraplaque macrophage and prevent the rupture of vulnerable plaques 10. Xu et al. indicated that in cardiac hypertrophy model, RORα could directly bind to the promotor region of MnSOD and transcriptionally activate expression of MnSOD, which further mediated the cardioprotective effects of melatonin 11. In the present study, we demonstrated that pressure overload might induce HF by down-regulation of RORα in the myocardium.

Inflammation has long been recognized as a key contributor of HF 12. Patel et al. indicated that CCR2^+^ monocyte-derived macrophage infiltrated the heart during the early phase of pressure-overload and played a vital role in the recruitment and activation of T cells 13. Laroumanie et al. revealed that CD4^+^ T cells were crucial for the progression from hypertrophy to HF. RAG2KO mice lacking T and B cells and MHCIIKO mice lacking CD4^+^ T cells did not develop ventricular dilation and dysfunction 14. Furthermore, Martini et al. used scRNA-seq to map the cardiac immune landscape in pressure overload-driven HF model 5. In this study, our scRNA-seq and scATAC-seq data indicated that B cells were significantly activated in the myocardium of failing hearts. Further analysis illustrating the functional heterogeneity of immune cell subtypes is needed.

ATAC-seq analysis revealed chromatin accessibility changes that precede the major transcriptional events and identified potential regulatory elements. Thus, subtle transition in cell state can be detectable before the changes in mRNA expression. Integrative analysis of ATAC-seq and RNA-seq data showed accessible promoters of marker genes involved in heart development and cell cycle regulation, including *Tnni3*, *Btg2,* and* Nr4a1*. The consistency between transcriptome and chromatin accessibility profiles reassured that the DEGs found in our study were truly derived from changes in the disease status.

Although bulk ATAC-seq provided new insights into the transcriptional reprogramming in HF, more detailed chromatin accessibility regulation in different types of cells can only be revealed through scATAC-seq. NO released from endothelial cells acts as a regulator in heart tissue with crucial autocrine/paracrine effects on cardiac function 15. Cardiac contractility is affected by NO which induces an earlier onset of relaxation, leading to a longer diastole and favoring diastolic filling. NO exerts multifactorial effects on various cell types in the heart and may play a role in growth of the vasculature and myocardial hypertrophy 16. NOS3, the synthase that catalyzes the production of biological NO from L-arginine, plays a critical role in heart development 17. By scATAC-seq, we identified increased chromatin accessibility of NOS3 in endothelial cells of failing hearts, indicating that elevated NO levels might be the results of increased NOS3 in HF.

In conclusion, we performed a comprehensive multi-omics analysis, comparing the transcriptomics, miRNA expression, and chromatin accessibility profile between the HF and control mice, thus providing mechanistic insights into the pathogenesis of pressure overload-induced HF.

## Supplementary Material

Supplementary figures.
