# Supplementary material for: A multi-omics approach to identify molecular alterations in a mouse model of heart failure
Source: Theranostics. 2022 Jan 16;12(4):1607–20. doi: 10.7150/thno.68232 (PMC8825596; doi:10.7150/thno.68232)
Supplement: Supplementary file 1 — Supplementary figures. [file thnov12p1607s1.pdf]

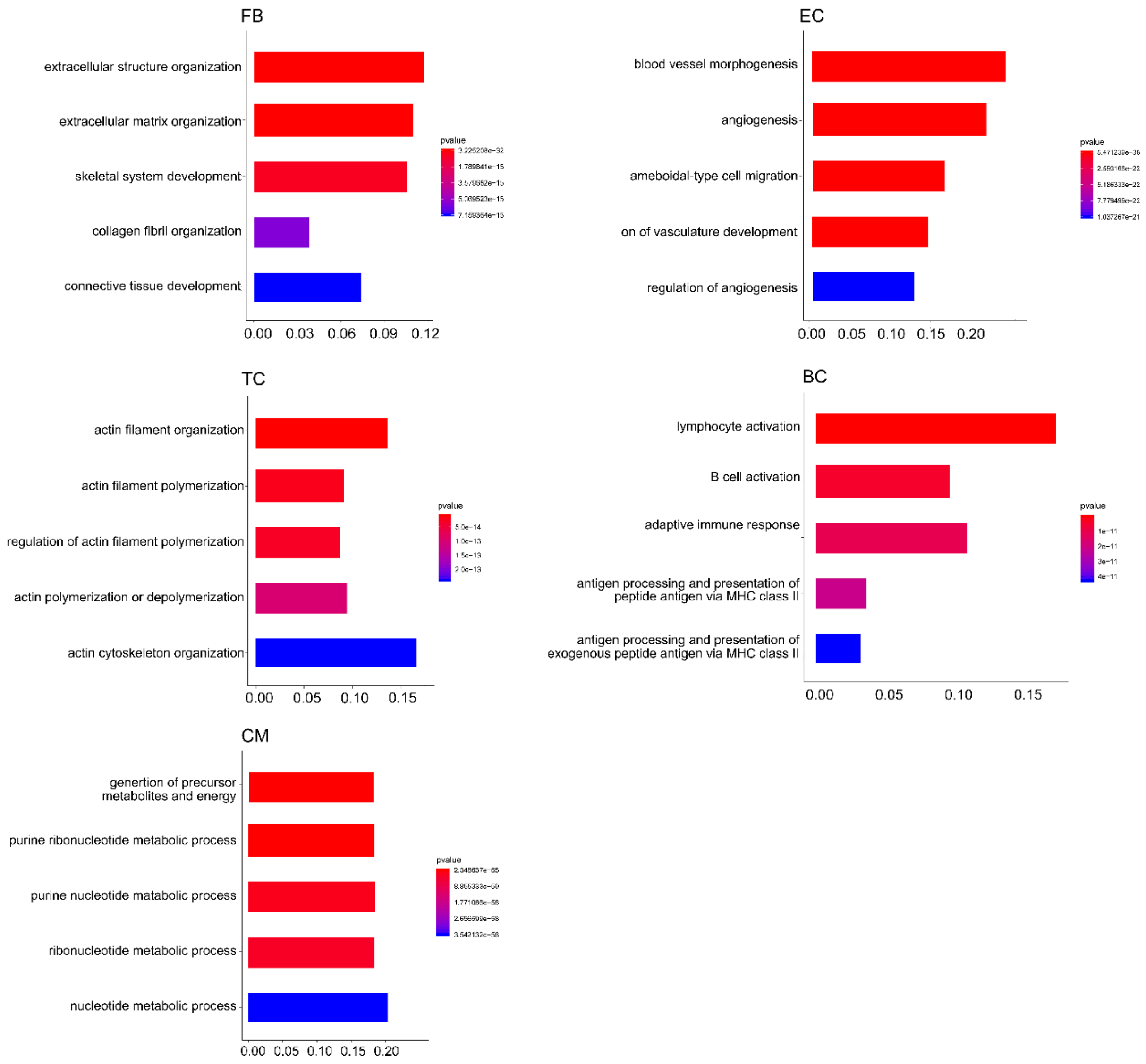

Figure S1. Gene ontology analysis of differentially expressed genes in each cell type.
FB: fibroblast; EC: endothelial cell; TC: T cell; BC: B cell; CM: cardiomyocyte.

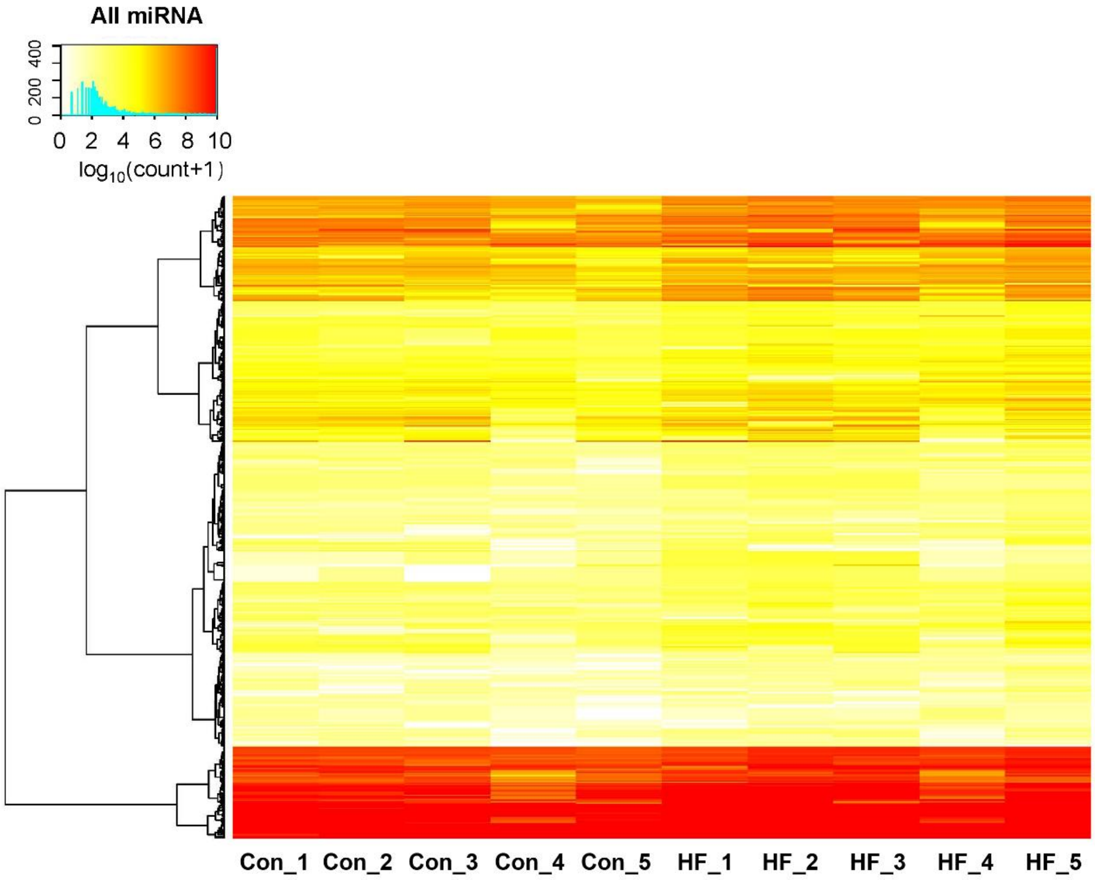

Figure S2. Heatmap of miRNA-seq analysis in control and heart failure (HF) mice.

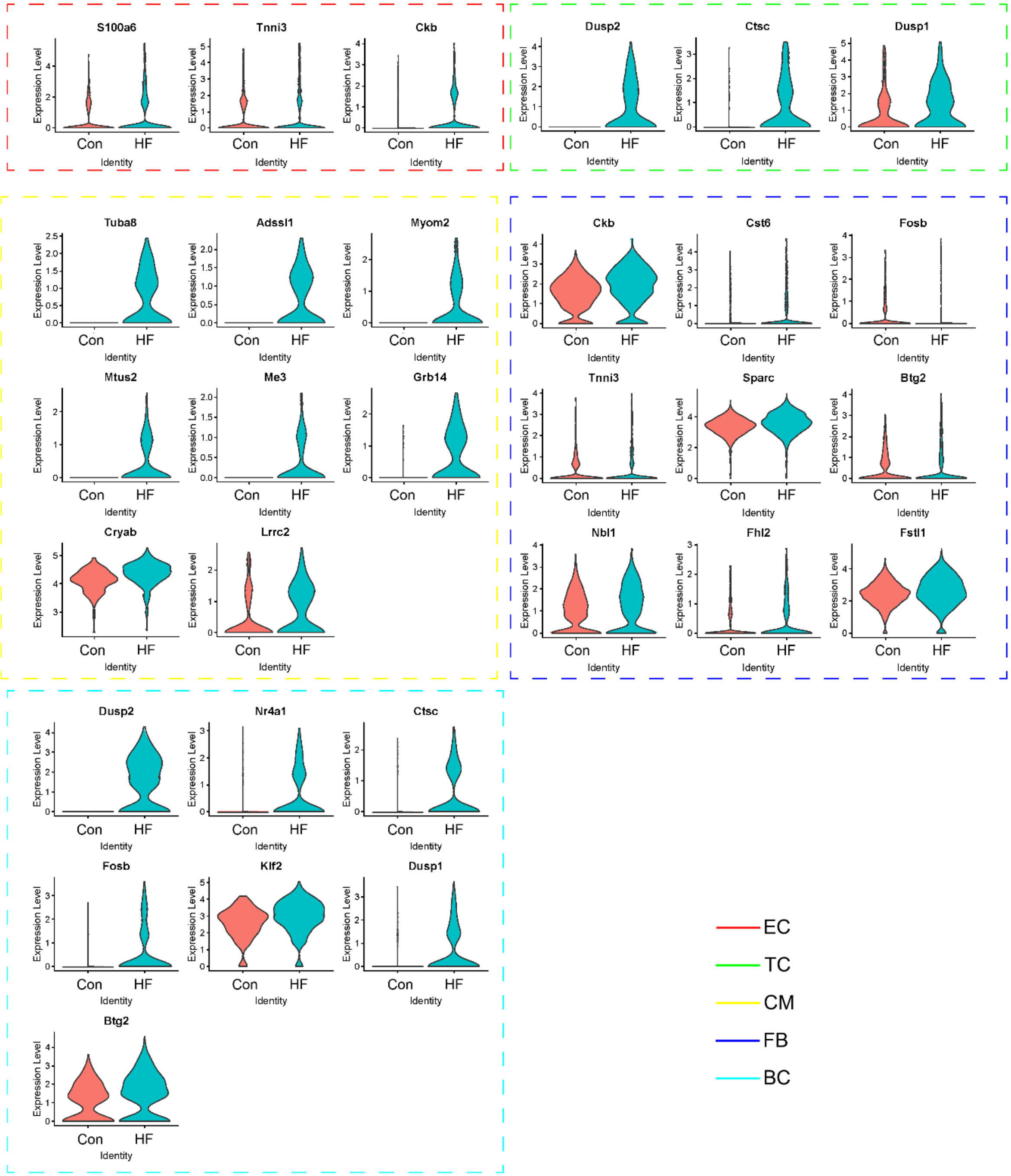

Figure S3. Upregulated genes in individual cell types of failing hearts with increased promotor accessibility. EC: endothelial cell; TC: T cell; CM: cardiomyocyte; FB: fibroblast; BC: B cell.

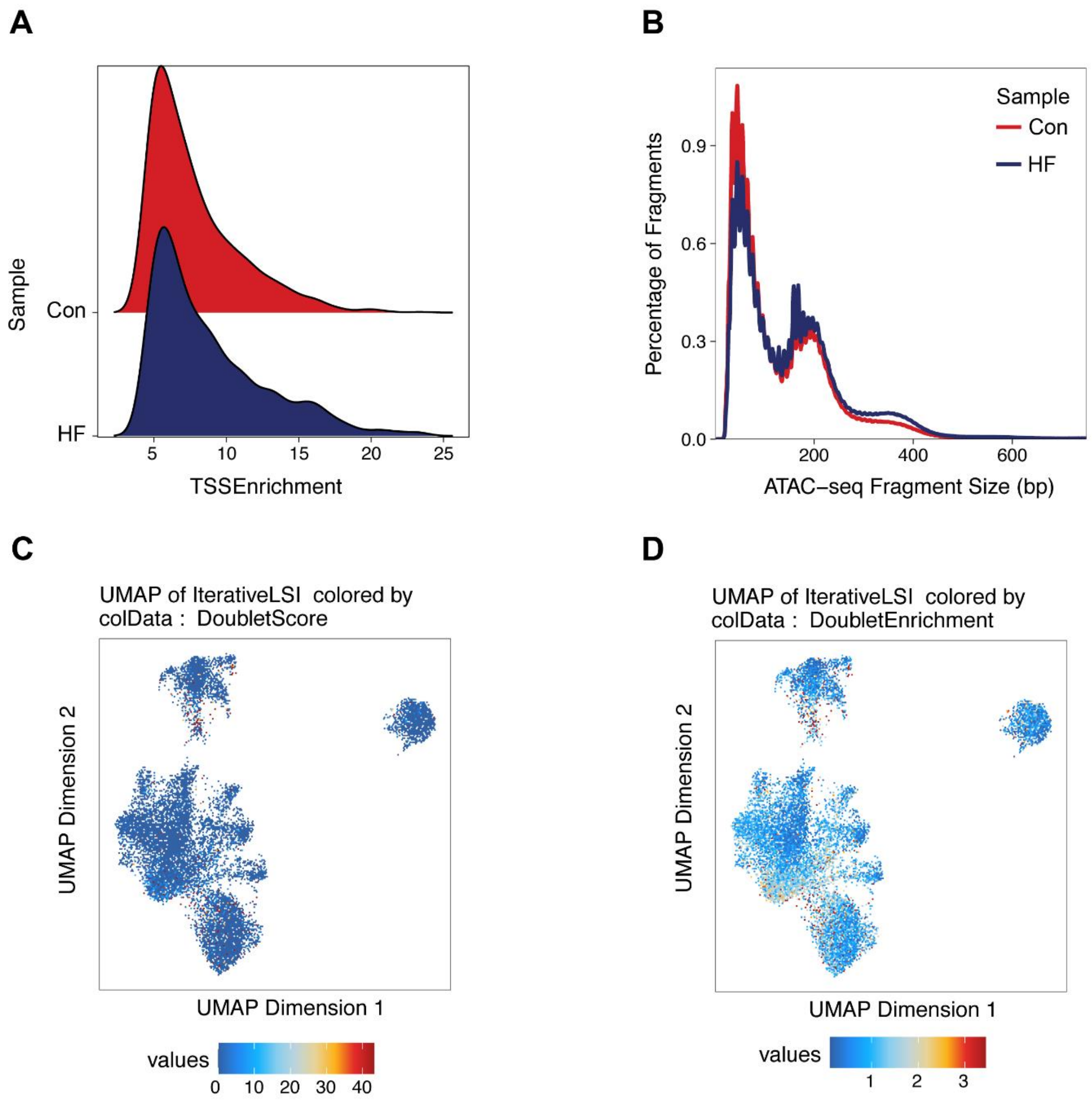

Figure S4. Quality control of scATAC-seq data. (A, B) The distribution of TSS enrichment and fragment size in control and heart failure (HF) mice**. (C, D)** The doublet score and doublet enrichment of cells passing filters.

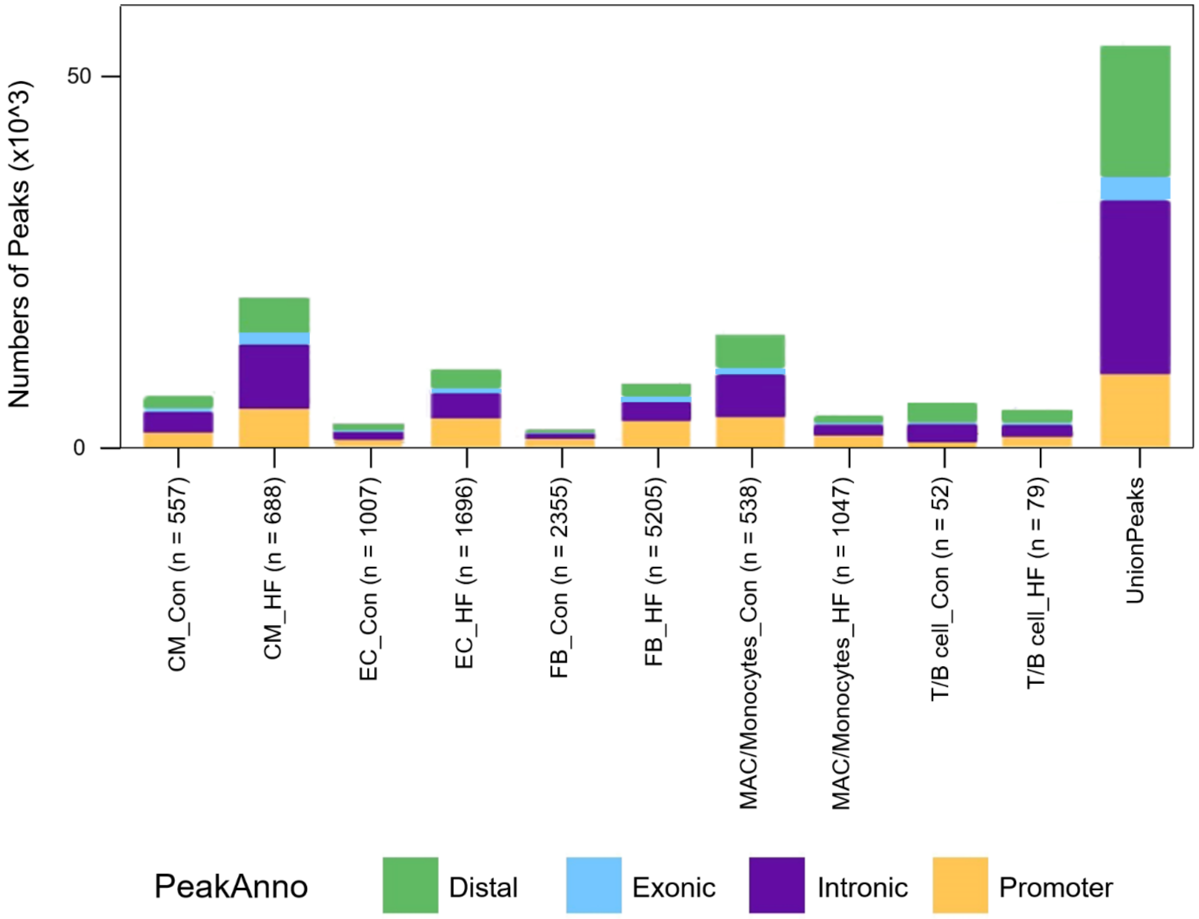

Figure S5. Peak calling of scATAC-seq data in control and heart failure (HF) samples.
